# Benzyl (*E*)-3-(2-methyl­benzyl­idene)dithio­carbazate

**DOI:** 10.1107/S1600536811033113

**Published:** 2011-08-27

**Authors:** Shang Shan, Zhao Wang, Yan-Lan Huang, Han-Qi Guo, Deng-Feng Li

**Affiliations:** a College of Chemical Engineering and Materials Science, Zhejiang University of Technology, People’s Republic of China

## Abstract

The title compound, C_16_H_16_N_2_S_2_, was obtained from the condensation reaction of benzyl dithio­carbazate and 2-methyl­benzaldehyde. The asymmetric unit contains two independent mol­ecules. In both mol­ecules, the methyl­phenyl ring and the dithio­carbazate fragment are located on opposite sides of the C=N bond, showing an *E* conformation. In each mol­ecule, the dithio­carbazate fragment is approximately planar, the r.m.s deviations being 0.018 and 0.025 Å. The mean plane of dithio­carbazate group is oriented at dihedral angles of 7.9 (3) and 68.24 (12)°, respectively, to the methyl­phenyl and phenyl rings in one mol­ecule, while the corresponding angles in the other mol­ecule are 10.9 (3) and 69.76 (16)°. Inter­molecular N—H⋯S hydrogen bonding occurs in the crystal structure to generate inversion dimers for both molecules.

## Related literature

For potential applications of hydrazone and its derivatives in the biological field, see: Okabe *et al.* (1993 ▶); Hu *et al.* (2001 ▶). For related structures, see: Shan *et al.* (2006 ▶, 2008*a* ▶,*b* ▶, 2011 ▶). For the synthesis, see: Hu *et al.* (2001 ▶). 
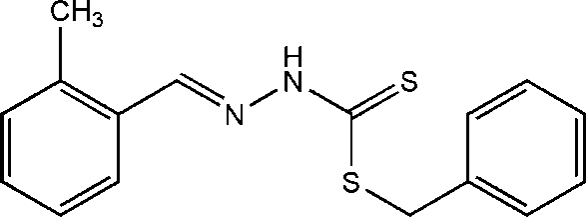


## Experimental

### 

#### Crystal data

C_16_H_16_N_2_S_2_
*M* *_r_* = 300.43
Monoclinic, 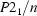

*a* = 21.976 (7) Å
*b* = 6.126 (3) Å
*c* = 23.099 (6) Å
β = 90.840 (4)°
*V* = 3109 (2) Å^3^
*Z* = 8
Mo *K*α radiation
μ = 0.33 mm^−1^
*T* = 294 K
0.29 × 0.23 × 0.18 mm

#### Data collection

Rigaku R-AXIS RAPID IP diffractometer
Absorption correction: multi-scan (*ABSCOR*; Higashi, 1995 ▶) *T*_min_ = 0.85, *T*_max_ = 0.93
11281 measured reflections
5596 independent reflections
2739 reflections with *I* > 2σ(*I*)
*R*_int_ = 0.055

#### Refinement

*R*[*F* ^2^ > 2σ(*F* ^2^)] = 0.065
*wR*(*F* ^2^) = 0.162
*S* = 1.02
5596 reflections
363 parameters
H-atom parameters constrained
Δρ_max_ = 0.26 e Å^−3^
Δρ_min_ = −0.19 e Å^−3^

### 

Data collection: *PROCESS-AUTO* (Rigaku, 1998 ▶); cell refinement: *PROCESS-AUTO* (Rigaku, 1998 ▶); data reduction: *CrystalStructure* (Rigaku/MSC, 2002 ▶); program(s) used to solve structure: *SIR92* (Altomare *et al.*, 1993 ▶); program(s) used to refine structure: *SHELXL97* (Sheldrick, 2008 ▶); molecular graphics: *ORTEP-3 for Windows* (Farrugia, 1997 ▶); software used to prepare material for publication: *WinGX* (Farrugia, 1999 ▶).

## Supplementary Material

Crystal structure: contains datablock(s) I, global. DOI: 10.1107/S1600536811033113/xu5276sup1.cif

Structure factors: contains datablock(s) I. DOI: 10.1107/S1600536811033113/xu5276Isup2.hkl

Supplementary material file. DOI: 10.1107/S1600536811033113/xu5276Isup3.cml

Additional supplementary materials:  crystallographic information; 3D view; checkCIF report

## Figures and Tables

**Table 1:** Hydrogen-bond geometry (Å, °)

*D*—H⋯*A*	*D*—H	H⋯*A*	*D*⋯*A*	*D*—H⋯*A*
N2—H2*N*⋯S1^i^	0.86	2.56	3.400 (4)	165
N4—H4*N*⋯S3^ii^	0.86	2.77	3.577 (4)	157

Symmetry codes: (i) 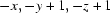; (ii) 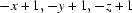.

**Fig. 1.**
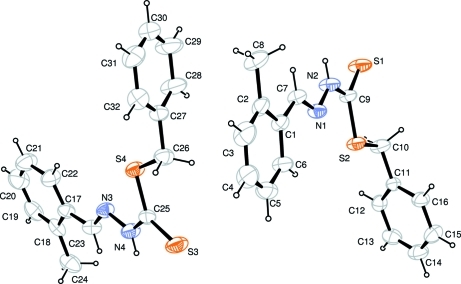
The molecular structure of the title compound with 40% probability displacement (arbitrary spheres for H atoms).

## supplementary crystallographic information

### Comment

Hydrazone and its derivatives have shown the potential application in the biological field (Okabe *et al.*, 1993; Hu *et al.*, 2001). As part of the ongoing investigation on anti-cancer compounds, the title compound has recently been prepared in our laboratory and its crystal structure is presented here.

The asymmetric unit of the title compound contains two independent molecules. In both molecules, the methylphenyl ring and dithiocarbazate fragment are located on the opposite sides of the C═N bond, showing the E-configuration. This agrees with those found in the structures reported previously (Shan *et al.*, 2006; Shan *et al.*, 2008*a*,*b*). In each molecule, the dithiocarbazate fragment is approximately planar, the r.m.s deviation being 0.0177 and 0.0248 Å, respectively. The mean plane of dithiocarbazate is oriented with respect to the methylphenyl and phenyl rings at 7.9 (3) and 68.24 (12)° in the C1-containing molecule; while the corresponding angles are 10.9 (3) and 69.76 (16)° in the other molecule.

Intermolecular N—H···S hydrogen bonding occurs in the crystal structure (Table 1).

### Experimental

Benzyl dithiocarbazate was synthesized as described previously (Hu *et al.*, 2001). Benzyl dithiocarbazate (0.4 g, 2 mmol) and 2-methylbenzaldehyde (0.24 g, 2 mmol) were dissolved in ethanol (20 ml), then acetic acid (0.2 ml) was added to the ethanol solution with stirring. The mixture solution was refluxed for 6 h. After cooling to room temperature, yellow microcrystals appeared. The microcrystals were separated from the solution and washed with cold water three times. Recrystallization was performed twice with absolute methanol to obtain single crystals of the title compound.

### Refinement

H atoms were placed in calculated positions with C—H = 0.93–0.97 Å and N—H = 0.86 Å, and refined in riding mode with *U*_iso_(H) = 1.5*U*_eq_(C) for methyl H atoms and 1.2*U*_eq_(C,N) for the others.

### Crystal data

**Table:**

C_16_H_16_N_2_S_2_	*F*(000) = 1264
*M**_r_* = 300.43	*D*_x_ = 1.284 Mg m^−^^3^
Monoclinic, *P*2_1_/*n*	Mo *K*α radiation, λ = 0.71073 Å
Hall symbol: -P 2yn	Cell parameters from 5596 reflections
*a* = 21.976 (7) Å	θ = 3.3–25.2°
*b* = 6.126 (3) Å	µ = 0.33 mm^−^^1^
*c* = 23.099 (6) Å	*T* = 294 K
β = 90.840 (4)°	Block, yellow
*V* = 3109 (2) Å^3^	0.29 × 0.23 × 0.18 mm
*Z* = 8	

### Data collection

**Table:**

Rigaku R-AXIS RAPID IP diffractometer	5596 independent reflections
Radiation source: fine-focus sealed tube	2739 reflections with *I* > 2σ(*I*)
graphite	*R*_int_ = 0.055
Detector resolution: 10.0 pixels mm^-1^	θ_max_ = 25.2°, θ_min_ = 3.4°
ω scans	*h* = −25→26
Absorption correction: multi-scan (*ABSCOR*; Higashi, 1995)	*k* = −6→7
*T*_min_ = 0.85, *T*_max_ = 0.93	*l* = −18→27
11281 measured reflections	

### Refinement

**Table:**

Refinement on *F*^2^	Primary atom site location: structure-invariant direct methods
Least-squares matrix: full	Secondary atom site location: difference Fourier map
*R*[*F*^2^ > 2σ(*F*^2^)] = 0.065	Hydrogen site location: inferred from neighbouring sites
*wR*(*F*^2^) = 0.162	H-atom parameters constrained
*S* = 1.02	*w* = 1/[σ^2^(*F*_o_^2^) + (0.0432*P*)^2^] where *P* = (*F*_o_^2^ + 2*F*_c_^2^)/3
5596 reflections	(Δ/σ)_max_ = 0.001
363 parameters	Δρ_max_ = 0.26 e Å^−^^3^
0 restraints	Δρ_min_ = −0.19 e Å^−^^3^

### Special details

**Table:**

Geometry. All e.s.d.'s (except the e.s.d. in the dihedral angle between two l.s. planes) are estimated using the full covariance matrix. The cell e.s.d.'s are taken into account individually in the estimation of e.s.d.'s in distances, angles and torsion angles; correlations between e.s.d.'s in cell parameters are only used when they are defined by crystal symmetry. An approximate (isotropic) treatment of cell e.s.d.'s is used for estimating e.s.d.'s involving l.s. planes.
Refinement. Refinement of *F*^2^ against ALL reflections. The weighted *R*-factor *wR* and goodness of fit *S* are based on *F*^2^, conventional *R*-factors *R* are based on *F*, with *F* set to zero for negative *F*^2^. The threshold expression of *F*^2^ > σ(*F*^2^) is used only for calculating *R*-factors(gt) *etc*. and is not relevant to the choice of reflections for refinement. *R*-factors based on *F*^2^ are statistically about twice as large as those based on *F*, and *R*- factors based on ALL data will be even larger.

### Fractional atomic coordinates and isotropic or equivalent isotropic displacement parameters (Å^2^)

**Table:**

	*x*	*y*	*z*	*U*_iso_*/*U*_eq_	
S1	0.00768 (5)	0.7325 (2)	0.57333 (6)	0.0829 (5)	
S2	0.12246 (5)	0.58916 (18)	0.63604 (5)	0.0611 (4)	
S3	0.42020 (6)	0.2494 (2)	0.49716 (6)	0.0866 (5)	
S4	0.35943 (5)	0.34105 (19)	0.38154 (6)	0.0652 (4)	
N1	0.12405 (14)	0.2604 (6)	0.55431 (16)	0.0584 (10)	
N2	0.07408 (15)	0.3932 (6)	0.54827 (17)	0.0663 (11)	
H2N	0.0474	0.3656	0.5217	0.080*	
N3	0.44164 (15)	0.6850 (6)	0.37635 (17)	0.0599 (10)	
N4	0.44538 (15)	0.5710 (6)	0.42774 (18)	0.0686 (11)	
H4N	0.4702	0.6145	0.4544	0.082*	
C1	0.17913 (18)	−0.0439 (6)	0.51748 (19)	0.0538 (11)	
C2	0.1794 (2)	−0.2255 (8)	0.4810 (2)	0.0660 (12)	
C3	0.2286 (3)	−0.3619 (9)	0.4846 (3)	0.0940 (18)	
H3	0.2294	−0.4847	0.4610	0.113*	
C4	0.2768 (3)	−0.3257 (9)	0.5215 (3)	0.0915 (18)	
H4	0.3094	−0.4223	0.5228	0.110*	
C5	0.2763 (2)	−0.1464 (9)	0.5561 (2)	0.0819 (16)	
H5	0.3091	−0.1190	0.5810	0.098*	
C6	0.22812 (18)	−0.0064 (7)	0.5549 (2)	0.0667 (13)	
H6	0.2280	0.1148	0.5791	0.080*	
C7	0.12811 (18)	0.1054 (7)	0.5170 (2)	0.0583 (12)	
H7	0.0976	0.0889	0.4890	0.070*	
C8	0.1282 (2)	−0.2723 (8)	0.4390 (2)	0.0920 (18)	
H8A	0.1301	−0.1726	0.4071	0.138*	
H8B	0.0900	−0.2550	0.4582	0.138*	
H8C	0.1316	−0.4193	0.4251	0.138*	
C9	0.06632 (17)	0.5648 (6)	0.58315 (19)	0.0535 (11)	
C10	0.0965 (2)	0.8266 (7)	0.6743 (2)	0.0695 (14)	
H10A	0.0980	0.9532	0.6492	0.083*	
H10B	0.0547	0.8056	0.6860	0.083*	
C11	0.13608 (16)	0.8637 (7)	0.72670 (19)	0.0491 (10)	
C12	0.17184 (18)	1.0460 (7)	0.7309 (2)	0.0603 (12)	
H12	0.1729	1.1440	0.7002	0.072*	
C13	0.2064 (2)	1.0868 (8)	0.7802 (3)	0.0735 (15)	
H13	0.2300	1.2126	0.7828	0.088*	
C14	0.2058 (2)	0.9446 (9)	0.8243 (2)	0.0819 (16)	
H14	0.2294	0.9705	0.8574	0.098*	
C15	0.1703 (2)	0.7607 (9)	0.8206 (2)	0.0826 (15)	
H15	0.1699	0.6613	0.8510	0.099*	
C16	0.13541 (19)	0.7239 (7)	0.7718 (2)	0.0659 (13)	
H16	0.1109	0.6004	0.7698	0.079*	
C17	0.48183 (18)	0.9804 (7)	0.3223 (2)	0.0614 (13)	
C18	0.51708 (19)	1.1720 (8)	0.3223 (3)	0.0715 (14)	
C19	0.5172 (2)	1.2945 (8)	0.2726 (3)	0.0870 (18)	
H19	0.5399	1.4227	0.2723	0.104*	
C20	0.4858 (3)	1.2379 (11)	0.2236 (3)	0.108 (2)	
H20	0.4871	1.3264	0.1909	0.130*	
C21	0.4525 (2)	1.0512 (10)	0.2228 (3)	0.102 (2)	
H21	0.4316	1.0090	0.1893	0.123*	
C22	0.4500 (2)	0.9238 (8)	0.2725 (2)	0.0798 (15)	
H22	0.4263	0.7980	0.2723	0.096*	
C23	0.4788 (2)	0.8437 (7)	0.3726 (2)	0.0664 (14)	
H23	0.5049	0.8723	0.4038	0.080*	
C24	0.5559 (2)	1.2373 (8)	0.3736 (3)	0.097 (2)	
H24A	0.5888	1.1352	0.3783	0.145*	
H24B	0.5317	1.2375	0.4078	0.145*	
H24C	0.5721	1.3808	0.3673	0.145*	
C25	0.41069 (17)	0.3917 (7)	0.43703 (19)	0.0565 (12)	
C26	0.31883 (18)	0.1063 (7)	0.4079 (2)	0.0691 (14)	
H26A	0.3464	−0.0166	0.4119	0.083*	
H26B	0.3022	0.1380	0.4457	0.083*	
C27	0.26839 (18)	0.0506 (7)	0.3661 (2)	0.0606 (12)	
C28	0.2196 (2)	0.1840 (9)	0.3593 (3)	0.103 (2)	
H28	0.2176	0.3112	0.3811	0.123*	
C29	0.1729 (3)	0.1357 (12)	0.3207 (3)	0.120 (3)	
H29	0.1400	0.2301	0.3168	0.144*	
C30	0.1750 (3)	−0.0472 (11)	0.2887 (3)	0.0939 (18)	
H30	0.1434	−0.0817	0.2631	0.113*	
C31	0.2229 (3)	−0.1785 (8)	0.2943 (2)	0.0888 (17)	
H31	0.2249	−0.3045	0.2719	0.107*	
C32	0.2703 (2)	−0.1306 (8)	0.3329 (2)	0.0776 (15)	
H32	0.3035	−0.2243	0.3359	0.093*	

### Atomic displacement parameters (Å^2^)

**Table:**

	*U*^11^	*U*^22^	*U*^33^	*U*^12^	*U*^13^	*U*^23^
S1	0.0705 (8)	0.0936 (9)	0.0833 (11)	0.0323 (7)	−0.0394 (8)	−0.0285 (8)
S2	0.0568 (6)	0.0694 (8)	0.0564 (8)	0.0151 (6)	−0.0207 (6)	−0.0124 (6)
S3	0.0834 (8)	0.1052 (11)	0.0703 (10)	−0.0175 (8)	−0.0289 (8)	0.0303 (8)
S4	0.0670 (7)	0.0727 (8)	0.0554 (8)	0.0018 (6)	−0.0150 (6)	0.0100 (6)
N1	0.0523 (19)	0.061 (2)	0.061 (3)	0.0101 (18)	−0.0145 (19)	−0.003 (2)
N2	0.060 (2)	0.069 (2)	0.070 (3)	0.0154 (19)	−0.027 (2)	−0.017 (2)
N3	0.065 (2)	0.058 (2)	0.056 (3)	−0.0019 (19)	−0.0104 (19)	0.015 (2)
N4	0.066 (2)	0.076 (3)	0.063 (3)	−0.003 (2)	−0.016 (2)	0.007 (2)
C1	0.062 (2)	0.050 (2)	0.049 (3)	0.013 (2)	−0.005 (2)	0.001 (2)
C2	0.078 (3)	0.075 (3)	0.044 (3)	0.010 (3)	0.001 (2)	−0.007 (3)
C3	0.116 (4)	0.086 (4)	0.080 (5)	0.032 (4)	0.005 (4)	−0.026 (3)
C4	0.096 (4)	0.093 (4)	0.086 (5)	0.034 (3)	0.005 (4)	−0.013 (4)
C5	0.067 (3)	0.098 (4)	0.080 (4)	0.016 (3)	−0.015 (3)	0.001 (3)
C6	0.063 (3)	0.071 (3)	0.065 (4)	0.012 (2)	−0.018 (2)	−0.011 (2)
C7	0.064 (3)	0.059 (3)	0.052 (3)	0.002 (2)	−0.014 (2)	−0.006 (2)
C8	0.116 (4)	0.096 (4)	0.063 (4)	0.005 (3)	−0.027 (3)	−0.026 (3)
C9	0.053 (2)	0.059 (3)	0.047 (3)	0.009 (2)	−0.016 (2)	−0.005 (2)
C10	0.072 (3)	0.075 (3)	0.061 (3)	0.019 (2)	−0.025 (3)	−0.021 (3)
C11	0.047 (2)	0.053 (2)	0.047 (3)	0.002 (2)	−0.004 (2)	0.001 (2)
C12	0.066 (3)	0.055 (3)	0.060 (3)	0.004 (2)	0.003 (2)	0.007 (2)
C13	0.066 (3)	0.065 (3)	0.088 (4)	−0.008 (3)	−0.011 (3)	−0.016 (3)
C14	0.079 (3)	0.094 (4)	0.071 (4)	0.003 (3)	−0.022 (3)	−0.020 (3)
C15	0.098 (4)	0.087 (4)	0.062 (4)	0.002 (3)	−0.009 (3)	0.016 (3)
C16	0.065 (3)	0.070 (3)	0.063 (3)	−0.017 (2)	−0.013 (3)	0.003 (3)
C17	0.057 (2)	0.058 (3)	0.069 (4)	0.009 (2)	−0.007 (2)	0.009 (3)
C18	0.047 (2)	0.065 (3)	0.103 (5)	0.000 (2)	0.016 (3)	0.005 (3)
C19	0.063 (3)	0.070 (4)	0.130 (6)	−0.013 (3)	0.022 (3)	0.020 (4)
C20	0.092 (4)	0.117 (5)	0.116 (6)	−0.018 (4)	0.007 (4)	0.042 (4)
C21	0.103 (4)	0.124 (5)	0.079 (5)	−0.027 (4)	−0.019 (4)	0.028 (4)
C22	0.088 (3)	0.078 (3)	0.072 (4)	−0.016 (3)	−0.009 (3)	0.014 (3)
C23	0.061 (3)	0.063 (3)	0.074 (4)	0.002 (2)	−0.017 (3)	0.001 (3)
C24	0.066 (3)	0.084 (4)	0.141 (6)	−0.013 (3)	−0.001 (4)	−0.021 (4)
C25	0.050 (2)	0.062 (3)	0.057 (3)	0.010 (2)	−0.007 (2)	0.008 (2)
C26	0.065 (3)	0.072 (3)	0.071 (4)	−0.003 (2)	−0.012 (3)	0.016 (3)
C27	0.059 (2)	0.061 (3)	0.062 (3)	0.006 (2)	−0.011 (2)	0.003 (3)
C28	0.102 (4)	0.112 (4)	0.093 (5)	0.044 (4)	−0.046 (4)	−0.045 (4)
C29	0.097 (4)	0.151 (6)	0.111 (6)	0.044 (4)	−0.053 (4)	−0.036 (5)
C30	0.082 (4)	0.123 (5)	0.076 (5)	−0.013 (4)	−0.013 (3)	−0.007 (4)
C31	0.120 (4)	0.069 (3)	0.077 (4)	−0.016 (3)	−0.006 (4)	−0.024 (3)
C32	0.086 (3)	0.061 (3)	0.085 (4)	0.008 (3)	0.000 (3)	0.000 (3)

### Geometric parameters (Å, °)

**Table:**

S1—C9	1.661 (4)	C13—C14	1.341 (7)
S2—C9	1.730 (4)	C13—H13	0.9300
S2—C10	1.800 (4)	C14—C15	1.372 (6)
S3—C25	1.651 (4)	C14—H14	0.9300
S4—C25	1.722 (4)	C15—C16	1.372 (6)
S4—C26	1.803 (4)	C15—H15	0.9300
N1—C7	1.287 (5)	C16—H16	0.9300
N1—N2	1.372 (4)	C17—C22	1.381 (6)
N2—C9	1.337 (5)	C17—C18	1.406 (6)
N2—H2N	0.8600	C17—C23	1.436 (6)
N3—C23	1.273 (5)	C18—C19	1.372 (7)
N3—N4	1.378 (5)	C18—C24	1.503 (7)
N4—C25	1.356 (5)	C19—C20	1.362 (7)
N4—H4N	0.8600	C19—H19	0.9300
C1—C6	1.390 (5)	C20—C21	1.357 (7)
C1—C2	1.396 (6)	C20—H20	0.9300
C1—C7	1.447 (5)	C21—C22	1.391 (7)
C2—C3	1.370 (6)	C21—H21	0.9300
C2—C8	1.502 (5)	C22—H22	0.9300
C3—C4	1.366 (6)	C23—H23	0.9300
C3—H3	0.9300	C24—H24A	0.9600
C4—C5	1.360 (7)	C24—H24B	0.9600
C4—H4	0.9300	C24—H24C	0.9600
C5—C6	1.362 (6)	C26—C27	1.500 (5)
C5—H5	0.9300	C26—H26A	0.9700
C6—H6	0.9300	C26—H26B	0.9700
C7—H7	0.9300	C27—C32	1.350 (6)
C8—H8A	0.9600	C27—C28	1.355 (6)
C8—H8B	0.9600	C28—C29	1.383 (6)
C8—H8C	0.9600	C28—H28	0.9300
C10—C11	1.497 (5)	C29—C30	1.343 (7)
C10—H10A	0.9700	C29—H29	0.9300
C10—H10B	0.9700	C30—C31	1.330 (7)
C11—C16	1.350 (6)	C30—H30	0.9300
C11—C12	1.368 (5)	C31—C32	1.391 (6)
C12—C13	1.381 (6)	C31—H31	0.9300
C12—H12	0.9300	C32—H32	0.9300
			
C9—S2—C10	100.88 (18)	C14—C15—H15	120.1
C25—S4—C26	102.3 (2)	C16—C15—H15	120.1
C7—N1—N2	115.6 (3)	C11—C16—C15	121.2 (4)
C9—N2—N1	120.9 (3)	C11—C16—H16	119.4
C9—N2—H2N	119.5	C15—C16—H16	119.4
N1—N2—H2N	119.5	C22—C17—C18	118.9 (5)
C23—N3—N4	114.5 (4)	C22—C17—C23	119.9 (4)
C25—N4—N3	121.3 (3)	C18—C17—C23	121.2 (4)
C25—N4—H4N	119.3	C19—C18—C17	117.6 (5)
N3—N4—H4N	119.3	C19—C18—C24	120.4 (5)
C6—C1—C2	119.9 (4)	C17—C18—C24	121.9 (5)
C6—C1—C7	119.6 (4)	C20—C19—C18	123.3 (5)
C2—C1—C7	120.6 (4)	C20—C19—H19	118.4
C3—C2—C1	117.3 (4)	C18—C19—H19	118.4
C3—C2—C8	120.5 (5)	C21—C20—C19	119.5 (6)
C1—C2—C8	122.2 (4)	C21—C20—H20	120.2
C4—C3—C2	122.9 (5)	C19—C20—H20	120.2
C4—C3—H3	118.5	C20—C21—C22	119.3 (5)
C2—C3—H3	118.5	C20—C21—H21	120.4
C5—C4—C3	119.0 (5)	C22—C21—H21	120.4
C5—C4—H4	120.5	C17—C22—C21	121.4 (5)
C3—C4—H4	120.5	C17—C22—H22	119.3
C4—C5—C6	120.6 (5)	C21—C22—H22	119.3
C4—C5—H5	119.7	N3—C23—C17	122.5 (4)
C6—C5—H5	119.7	N3—C23—H23	118.7
C5—C6—C1	120.2 (4)	C17—C23—H23	118.7
C5—C6—H6	119.9	C18—C24—H24A	109.5
C1—C6—H6	119.9	C18—C24—H24B	109.5
N1—C7—C1	121.5 (4)	H24A—C24—H24B	109.5
N1—C7—H7	119.3	C18—C24—H24C	109.5
C1—C7—H7	119.3	H24A—C24—H24C	109.5
C2—C8—H8A	109.5	H24B—C24—H24C	109.5
C2—C8—H8B	109.5	N4—C25—S3	119.8 (3)
H8A—C8—H8B	109.5	N4—C25—S4	113.0 (3)
C2—C8—H8C	109.5	S3—C25—S4	127.2 (3)
H8A—C8—H8C	109.5	C27—C26—S4	109.2 (3)
H8B—C8—H8C	109.5	C27—C26—H26A	109.8
N2—C9—S1	120.6 (3)	S4—C26—H26A	109.8
N2—C9—S2	113.4 (3)	C27—C26—H26B	109.8
S1—C9—S2	126.0 (3)	S4—C26—H26B	109.8
C11—C10—S2	109.6 (3)	H26A—C26—H26B	108.3
C11—C10—H10A	109.8	C32—C27—C28	117.4 (4)
S2—C10—H10A	109.8	C32—C27—C26	121.7 (4)
C11—C10—H10B	109.8	C28—C27—C26	120.9 (4)
S2—C10—H10B	109.8	C27—C28—C29	121.6 (5)
H10A—C10—H10B	108.2	C27—C28—H28	119.2
C16—C11—C12	118.4 (4)	C29—C28—H28	119.2
C16—C11—C10	121.0 (4)	C30—C29—C28	120.1 (5)
C12—C11—C10	120.5 (4)	C30—C29—H29	119.9
C11—C12—C13	121.0 (4)	C28—C29—H29	119.9
C11—C12—H12	119.5	C31—C30—C29	119.0 (5)
C13—C12—H12	119.5	C31—C30—H30	120.5
C14—C13—C12	119.8 (4)	C29—C30—H30	120.5
C14—C13—H13	120.1	C30—C31—C32	121.2 (5)
C12—C13—H13	120.1	C30—C31—H31	119.4
C13—C14—C15	119.8 (5)	C32—C31—H31	119.4
C13—C14—H14	120.1	C27—C32—C31	120.6 (5)
C15—C14—H14	120.1	C27—C32—H32	119.7
C14—C15—C16	119.8 (5)	C31—C32—H32	119.7

### Hydrogen-bond geometry (Å, °)

**Table:**

*D*—H···*A*	*D*—H	H···*A*	*D*···*A*	*D*—H···*A*
N2—H2N···S1^i^	0.86	2.56	3.400 (4)	165
N4—H4N···S3^ii^	0.86	2.77	3.577 (4)	157

Symmetry codes: (i) −*x*, −*y*+1, −*z*+1; (ii) −*x*+1, −*y*+1, −*z*+1.
